# Social Anxiety and Internet Addiction among Rural Left-behind Children: The Mediating Effect of Loneliness

**Published:** 2017-12

**Authors:** Yujia REN, Jiao YANG, Liqiong LIU

**Affiliations:** 1. Physical Education Institute, Hunan First Normal University, Changsha, Hunan, 410205, China; 2. School of Nursing, Central South University, Changsha, Hunan, 410013, China; 3. Xiangtan Vocational Technical College, Xiangtan, Hunan, 411005, China

**Keywords:** Rural left-behind children, Internet addiction, Loneliness, Social anxiety, Mediating effect

## Abstract

**Background:**

At present, the mental health of rural left-behind children is a major social problem in China. Internet addiction, social anxiety, and loneliness are common psychological and behavioral problems among rural left-behind children, but the relationships among these issues have not been clearly identified.

**Methods:**

A total of 432 junior year 1 to senior year 3 students were randomly selected from 2 rural middle schools in Hunan Province of China as the research subjects. The Internet Addiction Disorder Diagnostic Scale, Social Anxiety Subscale of the Self-Consciousness Scale (SASS-CS), and University of California Los Angeles Loneliness Scale were employed to measure the degree of Internet addiction, feelings of social fear, social avoidance behavior, and the level of loneliness among the research subjects.

**Results:**

The rate of Internet addiction among rural left-behind middle school students was 18.27%, and was correlated with the length of time their parents spent at home as well as whether one or both parents migrated for work. Positive correlations were found among Internet addiction, social anxiety, and loneliness. Loneliness was found to play a mediating role in the relationship between social anxiety and Internet addiction among rural left-behind middle school students.

**Conclusion:**

Social anxiety and loneliness both increase the likelihood of Internet addiction in rural left-behind middle school students and social anxiety can affect Internet addiction through loneliness, implying an urgent need to strengthen care for rural left-behind children, reduce their loneliness, and thereby effectively alleviate the problem of Internet addiction among rural left-behind middle school students.

## Introduction

Since their entry into the modern society of digitalization, networking, information, and globalization, human beings’ health has faced severe challenges. In the current cultural context, characterized by social changes and the rapid development of science and technology, mental health problems have become a serious issue that cannot be ignored in China (1). The high-speed development of the Internet and computer technology has brought unprecedented convenience to people’s lives, learning, and work. However, due to the unreasonable, unrestrained use of the Internet by many people, some negative influences of this technology have become increasingly apparent (2). Especially for young people, whose cognition is not fully developed, excessive use of the Internet may impair their studies and development, among other negative consequences. Internet addiction disorder (IAD) has become a widespread psychological and behavioral problem among teenagers (3).

Currently, the mental health of rural left-behind children is a pressing social problem. Left-behind children are a group generated under the special circumstances of Chinese society. The term mainly refers to juveniles who are left behind in their parents’ registered permanent residence and cannot live together with their parents, who are working in another place (4). This unusual family environment and growth experience has a number of negative effects on the psychology and behaviors of left-behind children. Rural left-behind children’s impaired self-awareness, dysfunctional relationships with their parents, and imbalanced social environment have contributed to the growing risk of Internet addiction (5). Through a large-scale investigation on 3,416 children in rural areas, researchers (6) have found that the rate of Internet addiction is significantly higher among rural left-behind children, especially middle school students, than among ordinary rural children. As a result, preventing and solving the problem of Internet addiction has become a top-priority task for middle school education. In-depth exploration of the factors that affect Internet addiction among left-behind children and the mechanism of interactions between different factors can provide a new perspective and new ideas for the prevention and control of Internet addiction among left-behind middle school students in rural areas.

Among a large number of factors that contribute to Internet addiction, anxiety and loneliness are two major factors, and Internet addiction can also exacerbate the addicts’ anxiety and loneliness (7). Researchers have found that adolescents’ social anxiety is strongly correlated with Internet addiction, but this relationship is regulated by extroverted personality traits (8). Social anxiety levels in adolescents are highly useful for predicting Internet addiction (9), and adolescents’ loneliness is significantly positively correlated with Internet addiction (10).

In summary, social anxiety and loneliness both affect Internet addiction among left-behind middle school students, and social anxiety can also positively predict loneliness (11, 12). Nonetheless, the relationships among social anxiety, loneliness, and Internet addiction in rural left-behind children have not yet been elucidated, and clarifying these relationships is the focus of this study. Using structural equation modeling, this study aims to explore the relationships among Internet addiction, social anxiety, and loneliness in rural left-behind middle school students. We also examine the mediating effect of loneliness on the relationship between social anxiety and Internet addiction among left-behind middle school students.

The ultimate aim of this research was to provide a reference and suggestions for the prevention and control of Internet addiction and the improvement of mental health among left-behind middle school students in rural areas.

## Methods

Overall, 432 left-behind middle school students were randomly selected from 2 rural middle schools in Hunan Province of China. Questionnaires were distributed to all of the students, and the students were guided to correctly fill out the questionnaire anonymously. A total of 416 valid copies of the questionnaire (211 from boys and 205 from girls) were recovered, with a valid response rate of 96.3%. The demographics of the subjects can be seen in Table 1.

**Table 1:** General characteristics of the subjects and the rate of Internet addiction

***Objects***		***Total number***	***Number of Internet addicts***	***Internet addiction rate (%)***	***χ^2^***	***P***
Gender	Male	211	50	23.70	8.447	0.004
	Female	205	26	12.68		
Only child	Yes	244	47	19.26	0.390	0.532
	No	172	29	16.86		
Grade	Junior 1	63	5	7.94	5.424^*^	0.020
	Junior 2	69	9	13.04		
	Junior 3	68	14	20.59		
	Senior 1	67	16	23.88		
	Senior 2	79	18	22.78		
	Senior 3	70	14	20.00		
Length of time parents spend at home	Less than 1 month	209	47	22.49	5.411^*^	0.020
	1–2 months	128	20	15.63		
	2–6 months	47	6	12.77		
	More than months	32	3	9.38		
Parents migrating for work	Father migrating for work	152	20	13.16	7.065	0.029
	Mother migrating for work	96	15	15.63		
	Both parents migrating for work	168	41	24.40		
Total			76	18.27		

* Indicates the use of the chi-square test for trend

The methodology and purpose of the survey were explained to the students as well as their teachers and parents, and the students’ consent was obtained before beginning the survey. All of the participants volunteered to participate in the survey. All participants were physically healthy, excluding genetic diseases and mental illnesses. The study was approved by Ethics Committee of the university.

### Measuring Tools

1. The Basic Situation Scale, compiled by the researchers, consists of 5 questions regarding gender, grade, whether the child is an only child, the length of time the child’s parents spend at home every year, and the parents’ migration for work.
2. The Internet Addiction Disorder Diagnostic Scale for Middle School Students (IADDS) used in this study was compiled by Zan, et al. (13) in strict accordance with psychometric standards, specifically item response theory (IRT). The scale includes a total of 13 items covering 3 dimensions: Internet craving and tolerance, adverse consequences, and withdrawal reactions. Two-level scoring is used, with “yes” scored 1 point and “no” scored 0 points. A total score ≥ 5 is classified as Internet addiction, and a score < 5 is classified as non-Internet addiction. The scale has shown good validity and reliability, and it consists of concise items in refined language, demonstrated to be in line with the psychological and linguistic characteristics of middle school students. The scale is easy to complete and suitable for the clinical diagnosis of Internet addiction among Chinese middle school students.
3. The ULS-8 Loneliness Scale was adapted from the UCLA-20 scale by Hays and DiMatteo (15). It contains a total of 8 items, including 6 positively scored “loneliness” items and 2 reverse-scored “non-loneliness” items (i.e., Item 3, “I am a person who is willing to make friends,” and Item 6, “When I am sad, I can find someone to accompany me”). Each item has 4 answer options: never (scored as 1 point), rarely (2 points), sometimes (3 points), and always (4 points). The positive statement (i.e., non-loneliness) items are scored using reverse-sequence scoring. The total score on the scale can range from 8 to 32 points, with a higher total score indicating a higher degree of loneliness. The 8 items of the scale support the single-factor model (15).
4. The SASS-CS (16), contains 6 items rated on a scale from 0 to 4 (0 = very inconsistent; 4 = very consistent). While the scale is intended for social anxiety measurement, the items measure not only subjective anxiety, but also speech and behavioral difficulties. The occasions described in the scale include unfamiliar occasions, being watched, embarrassing events, talking to strangers, public speaking, and being in a large group of people. The Social Anxiety Subscale was generated during the preparation of the Self-Consciousness Scale. The total score on the scale can range from 0 to 24 points, with a higher score indicating a higher degree of anxiety.

### Statistical Methods

The data were analyzed using SPSS 15.0 statistical software (Chicago, IL, USA). Cross-group comparisons of qualitative data were conducted using a chi-square test or chi-square test for trend. Cronbach’s *α* coefficients were used to measure the internal consistency of the questionnaire. Structural equation modeling was used to analyze the structural validity of the scales and the mediating effect. *P* < 0.05 was considered statistically significant.

## Results

### General Characteristics of the Subjects and Rate of Internet Addiction

Among the 416 valid questionnaires collected in this study, 76 Internet addicts were identified (18.27% of the sample). Notably, significantly more male Internet addicts were identified than female Internet addicts (*P*<0.001). Additionally, the rate of Internet addiction increased with increasing grade level (*P*<0.05). Moreover, the shorter the amount of time the parents spent at home, the higher the Internet addiction rate (*P*<0.001), and the rate of Internet addiction was higher among participants who had both parents migrating for work than those who only had one parent migrating for work (*P*<0.05). The rate of Internet addiction was also higher in only children than in non-only children, but the difference was not statistically significant (Table 1).

### Analysis of Reliability and Validity

In this study, the Cronbach’s *α* coefficient was used to measure the internal consistency of the questionnaire. The results of the analysis showed that the Cronbach’s *α* coefficients of all structural dimensions of the scale were higher than the minimum acceptable level of 0.7 (Table 2), indicating sufficient internal consistency of the questionnaire used in this study.

**Table 2:** Results of the reliability analysis of the survey scales in the questionnaire

**Scale**	**Dimension**	**Cronbach's** *α* **of each dimension**	**Total Cronbach's** *α*
IADDS	Internet craving and tolerance	0.721	0.712
	Adverse consequences	0.715	
	Withdrawal reactions	0.702	
SASS-CS		0.720	0.720
ULS-8 Loneliness Scale		0.797	0.797

The validity of the data collected from the questionnaire was analyzed by constructing a confirmatory factor analysis model. From the results shown in Table 3, we can see that each fitting index is below the reference value, suggesting that all 3 scales have good validity.

**Table 3:** Results of analyzing the validity of each scale

***Index***	***Reference value***	***IADDS***	***SASS-CS***	***ULS-8 Loneliness Scale***
χ^2^/df	< 3	1.639	1.763	1.853
GFI	> 0.90	0.944	0.931	0.921
CFI	> 0.90	0.980	0.952	0.932
NFI	> 0.90	0.952	0.932	0.922
RMSEA	< 0.080	0.079	0.062	0.067
IFI	> 0.90	0.982	0.952	0.932
AGFI	> 0.80	0.880	0.875	0.860

### Analysis of the Correlations among Internet Addiction, Social Anxiety, and Loneliness

As seen in Table 4, the correlation coefficient for social anxiety and Internet addiction was 0.385, the correlation coefficient for loneliness and Internet addiction was 0.324, and the correlation coefficient for loneliness and social anxiety was 0.540. Therefore, social anxiety, loneliness, and Internet addiction are mutually significantly positively correlated (*P* < 0.05).

**Table 4:** Analysis of the correlation between Internet addiction, social anxiety, and loneliness

	***Internet addiction***	***Social anxiety***	***Loneliness***
Internet addiction	1		
Social anxiety	0.385^**^	1	
Loneliness	0.324^**^	0.540^**^	1

** *P* < 0.01

### Analysis of Mediating Effect

**Fig. 1:**
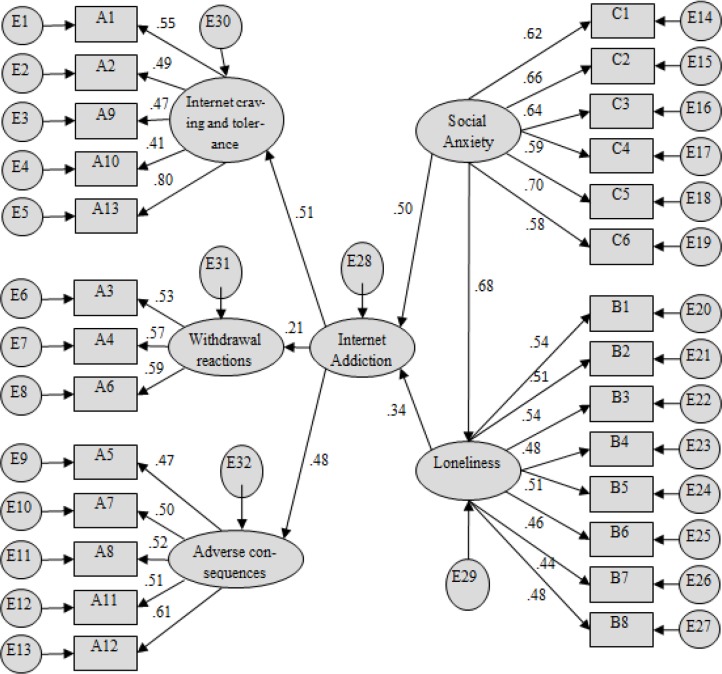
The mediating effect of loneliness on the relationship between social anxiety and Internet addiction (standard coefficients)

The mediation model was constructed according to the theoretical assumptions put forward in the introduction (Fig. 1). In this model, social anxiety was an exogenous observation variable, Internet addiction was an endogenous variable, and loneliness was a mediating variable. The test results of the 3 main paths of the model are shown in Table 5. All 3 paths were statistically significant, indicating that loneliness plays a mediating role in the relationship between social anxiety and Internet addiction among left-behind middle school students. Specifically, the direct effect of social anxiety on Internet addiction among left-behind middle school students was 0.50, and the indirect effect was 0.68 × 0.34 = 0.23. For the model fitting index, *X*^2^*/DF* = 1.16 was below the acceptable minimum value of 5, indicating that the results of the analysis are acceptable. *RMSEA* (root mean square error of approximation) = 0.020, *NFI* (normed fit index) = 0.922, *CFI* (comparative fit index) = 0.923, *IFI* (incremental fit index) = 0.972, *GFI* (goodness-of-fit index) = 0.939, *AGFI* (adjusted goodness-of-fit index) = 0.928, and *RFI* (relative fit index) = 0.910, which is greater than 0.9 and reaches the standard of the fitting index, indicating that the degree of fit between the model and the data is within the acceptable range.

**Table 5:** Test results of the main path parameters of the model

***Path***	***Non-standard coefficient***	***Standard error***	***CR***	***P***
Loneliness <--- Social anxiety	0.517	0.069	7.455	< 0.001
Internet addiction <--- Social anxiety	0.144	0.046	3.099	0.002
Internet addiction <--- Loneliness	0.13	0.061	2.111	0.035

## Discussion

### General Characteristics of the Research Subjects and Rate of Internet Addiction

We found that the rate of Internet addiction among rural left-behind children surveyed was as high as 18.27%, significantly higher than the rates found earlier (17, 18). However, those studies focused on Internet addiction among ordinary teenagers, with the rates of Internet addiction varying from 3.6% to 11.5%. The large differences among the results may be related to the scales used and sampling specificity in different studies and could also be related to demographic variables such as region, school type, and grade level. The Internet addiction rate found in this study is also higher than that of Jin et al., (6) who also studied rural left-behind children and obtained a rate of 6.83%. The reason for this difference is closely related to the living environment in which children were left behind. Jin’s subjects were left-behind children in poverty-stricken areas. Presumably, due to the limited economic development level, the availability of the Internet in these areas is not high. At the same time, children in these areas are often engaged in housework or farming activities to relieve the family burden, so they have little spare time, resulting in a lower likelihood of Internet addiction.

We also found that Internet addiction was significantly more prevalent among male students than among female students, which is consistent with the findings of other researchers (19, 20). This may be related to the psychological differences between males and females. In contrast to girls, boys are more likely to pursue more excitement and challenges. They are more independent and have more courage to try new things. On the other hand, boys have poor self-control and tend to indulge in the Internet, resulting in Internet addiction (19). The rate of Internet addiction among left-behind children also varied significantly with grade level. Between the junior 1 and senior 1 grade levels, the rate of Internet addiction increased, but the rate at the senior 3 level was lower than that at senior 1 and senior 2. There are a number of possible reasons for this. On the one hand, in the junior high school period, children are characterized by immature psychological development and low independence. On the other hand, junior high schools in rural areas are often closer to home, so the children are subject to supervision by other elders in the family. Furthermore, since junior high school students are required to complete a lot of homework, they do not have more opportunities to access the Internet, thereby reducing the possibility of Internet addiction. By contrast, during the senior high school period, students are usually studying far away from home. The lack of effective supervision by guardians may lead to an increased rate of Internet addiction among senior high school students. However, due to the approaching college entrance examination, students in senior year 3 face a great deal of studying, heavy pressure, and more supervision by their parents and teachers. As a result, the rate of Internet addiction is relatively low compared to those in senior years 1 and 2 (21).

Our study also found that longer time spent at home by parents each year was correlated with a lower rate of Internet addiction among left-behind children. Ecological system theory posits that the micro-system factors which impact individual physical and mental development are very important for the development of healthy psychological function in the individual (22). In particular, the family, especially the parents, is an extremely significant micro-system factor for the development of middle school students’ mental health. For example, parents give warmth and love in the process of getting along with middle school students, and through their own words and deeds can teach children to behave according to their expectations. In order to receive their parents’ recognition, children strive to meet their parents’ expectations of behaviors (23). Hence, left-behind middle school students are more eager to obtain their parents’ concern and love and are consequently more likely to reduce the behaviors that their parents do not recognize, such as surfing the Internet, in hope that their parents will spend more time with them (24), leading to a lower rate of Internet addiction among left-behind children with parents who spend more time at home.

### Correlation between Internet Addiction, Social Anxiety, and Loneliness

According to the results of our correlation analysis, there are positive correlations between social anxiety and Internet addiction, between loneliness and Internet addiction, and between social anxiety and loneliness. In terms of the definition of Internet addiction, previous research pointed out that excessive enthusiasm to establish interpersonal relationships through the Internet or to get interpersonal support from the Internet is one of the important manifestations of Internet addiction (25). The relationship between social anxiety and Internet addiction can be seen indirectly from this definition. Our findings are similar to those of Li et al. (26). The probable reasons for the correlation between social anxiety and Internet addiction include the lack of parents’ companionship with and guidance over left-behind middle school students, inappropriate education by the custodians whose cultural and teaching quality level is relatively low, the low level of rural education, and other factors which result in the tendency of left-behind middle school students to suffer from social anxiety. When the level of social anxiety increases and left-behind middle school students cannot find a reasonable outlet, the Internet naturally becomes an outlet of their inner anxiety. The virtual and convenient Internet can comfort left-behind middle school students when they face frustrations in the real world, improve their sense of social control, and enhance their self-confidence. Hence, social anxiety increases the incidence of Internet addiction. On the other hand, due to the long-term lack of parental role, left-behind middle school students cannot receive timely encouragement, support, and help from their parents when they encounter setbacks and problems in the process of growth, resulting in more intense feelings of loneliness (25). However, through the Internet, left-behind middle school students can find “like-minded” friends to share their troubles with, so as to reduce their inner sense of loneliness. Consequently, the intense sense of loneliness among left-behind middle school students increases the likelihood of Internet addiction. Moreover, when left-behind middle school students encounter a social setback in real life and cannot maintain good relationships with other peers, or perhaps are even excluded by other students, the absence of reasonable guidance or correct education by their parents would reinforce their tendency to lock away their inner world to avoid being hurt again (26). At the same time, we found that there is a positive correlation between social anxiety and loneliness in left-behind middle school students, and a previous study found that higher levels of social anxiety and loneliness result in higher trust in the Internet by individuals and a more positive attitude toward social networking [24]. In sum, excessive social anxiety brings left-behind middle school students a sense of loneliness along with a corresponding increase in the likelihood of Internet addiction.

### Mediating Effect of Loneliness on the Relationship between Social Anxiety and Internet Addiction among Left-Behind Middle School Students

The results of this study also showed that social anxiety can affect the rate of Internet addiction among left-behind middle school students both directly and through the partial mediating effect of loneliness. Left-behind middle school students may have self-attribution of their parents’ migration for work, and this attribution may also be generalized to other life situations. This cognitive bias would lead left-behind children to experience more negative emotions in social interaction situations, resulting in higher levels of social anxiety. The emergence of social anxiety would in turn strengthen the negative self-perception of left-behind middle school students, reducing their willingness, initiative, and self-confidence in their social relations and resulting in a decline in the quality of their interpersonal relationships and an increase in their loneliness (27). Social networking, on the other hand, is extremely open and inclusive. In order to reduce their level of inner anxiety and disperse their sense of loneliness, left-behind middle school students are likely to engage in social activities via the Internet, obtain social support from the virtual world, and thereby enhance their self-efficacy. Notably, long-term addiction to this increases the likelihood of Internet addiction (24).

Furthermore, according to Maslow’s Hierarchy of Needs, individual needs can be divided into 5 types of needs: physiological needs, safety needs, love and belonging needs, esteem needs, and self-actualization needs (28). An individual can only consider the satisfaction of higher-level needs once the lower-level needs have been satisfied. For left-behind middle school students, their basic physiological needs and safety needs may be met, but due to the lack of emotional connection with their parents and social anxiety caused by the difficulty getting along well with other peers, their love and belonging needs cannot be met. Over time, left-behind middle school students develop a strong sense of loneliness, and in order to alleviate this sense of loneliness, they are likely to find friends through the Internet in order to obtain social support and diminish their sense of loneliness, resulting in excessive use of the Internet (5).

In summary, rural left-behind middle school students experience increased social anxiety as a result of their unusual family environment. This social anxiety increases the students’ loneliness. In order to reduce their sense of loneliness, the students may seek out virtual social networking, thus increasing the possibility of Internet addiction.

## Conclusion

Social anxiety can affect the rate of Internet addiction among rural left-behind middle school students by influencing their loneliness. Internet addiction should be addressed from 3 perspectives to improve the mental health of rural left-behind children: 1) The main caregivers should give more care and guidance to rural left-behind children so that they can acquire more psychological support from the family when they face social frustrations. 2) Schools should pay more attention to the issue of peer interactions among left-behind middle school students, provide them proper guidance, and help them establish good interpersonal relationships, develop social skills, and relieve loneliness. 3) Schools should cooperate with the family and provide more care for rural left-behind children in order to reduce their loneliness and promote their mental health.

## Ethical considerations

The authors have completely observed the ethical issues (Including plagiarism, Informed Consent, misconduct, data fabrication and/or falsification, double publication and/or submission, redundancy, etc.).
